# Sandwich, Triple-Decker and Other Sandwich-like Complexes of Cyclopentadienyl Anions with Lithium or Sodium Cations

**DOI:** 10.3390/molecules27196269

**Published:** 2022-09-23

**Authors:** Sławomir J. Grabowski, Rubén D. Parra

**Affiliations:** 1 Polimero eta Material Aurreratuak: Fisika, Kimika eta Teknologia, Kimika Fakultatea, Euskal Herriko Unibertsitatea UPV/EHU & Donostia International Physics Center (DIPC) PK 1072, 20080 Donostia, Spain; 2 Ikerbasque, Basque Foundation for Science, 48011 Bilbao, Spain; 3 Department of Chemistry and Biochemistry, DePaul University, Chicago, IL 60614, USA

**Keywords:** sandwich complex, triple-decker complex, DFT calculations

## Abstract

Density functional theory, DFT, calculations were carried out on complexes containing cyclopentadienyl anions and lithium or sodium cations; half-sandwich, sandwich and sandwich-like complexes (among them triple-decker ones) are analyzed. Searches performed through the Cambridge Structural Database revealed that crystal structures containing these motifs exist, mostly structures with sodium cations. The DFT calculations performed here include geometry optimization and frequency calculations of the complexes at the ωB97XD/aug-cc-pVTZ level, followed by the partitioning of the energy of interaction via the Energy Decomposition Analysis scheme, EDA, at the BP86-D3/TZ2P level. Additional calculations and analyses were performed using both the Quantum Theory of Atoms in Molecules, QTAIM, and the Natural Bond Orbital analyses, NBO. The results of this work show that the electrostatic interaction energy is the most important attractive contribution to the total interaction energy of each of the complex systems analyzed here, and that complexation itself leads to minor electron charge shifts.

## 1. Introduction

The cyclopentadienyl anion is a species that is important in numerous reactions and processes of organometallic chemistry [1,2,3]. As a planar system containing six π-electrons, the cyclopentadienyl anion possesses properties typical of other aromatic systems [4,5]; its importance in the processes of catalysis is also very well-known. There are numerous energetically stable complexes and other units containing one or more cyclopentadienyl anions. Among the stable complexes, one can mention sandwich and half-sandwich species [6]. Ferrocene is a very well-known example of a sandwich complex [7]. The structure of ferrocene was proposed independently by Fischer and Pfab on the one hand [8] and by Wilkinson and co-workers on the other hand [9]. The actual crystal structure of ferrocene was solved by Dunitz and co-workers [10]. The authors of the latter study proposed the name “sandwich compounds” for such classes of systems [10].

The studies on ferrocene and on similar compounds have initiated extended investigations in organometallic chemistry on similar structures. One can mention, for example, the analysis of the process of proton transfer to the transition metal attached to cyclopentadienyl ion [11], the study on reactions of cyclopalladation and synthesis of borohydrides or the analysis of crystal structures of metal sandwich systems [12]. In the latter study, a special attention was paid to silver species. Other interesting investigations were performed on the sandwich-like structures’ complexes of corannulene with lithium and cesium cations [13]. Numerous other studies may be mentioned.

In this study, however, the emphasis is put on the nature of interactions in such classes of compounds, i.e., in sandwich-like species. This is why the results of theoretical calculations reported in former studies may be mentioned here. For example, high-level BP86/TZP geometry optimizations of the main-group metallocenes were performed, and such optimized geometries were then used for coupled-cluster theory energy calculations up to the CCSD(T) level [14]. Several findings and conclusions were presented in the latter study. In particular, it was found that the C_5_H_5_^−^-Be^2+^-C_5_H_5_^−^ complex structure of C_s_ symmetry is energetically more stable than the D_5d_ symmetry structure by about 0.6 kcal/mol. It was also found that for the C_5_H_5_^−^-Mg^2+^-C_5_H_5_^−^ complex, the structures of D_5h_ and D_5d_ symmetries correspond to energy minima and that their energy difference is negligible (0.01 kcal/mol). Energy partitioning analysis was performed on the mentioned sandwich structures. For the magnesium complex, the electrostatic and orbital interaction energies make up 71.6% and 28.4% of the attractive part of the interaction energy, respectively, whereas for the beryllium moiety these terms contribute 59.2% and 40.8% of the total attractive energy, respectively. The orbital interaction energy is related to the electron charge shifts, and consequently to the covalent character of an interaction. Accordingly, a greater importance of the covalency for the Be-cyclopentadienyl contacts was observed than that for the Mg-cyclopentadienyl ones. Similarly, for other systems, the more covalent and less ionic character of the beryllium species compared to their magnesium counterparts occurs. The latter conclusion is confirmed by other studies; a review on structures of metallocenes was performed, particularly an emphasis was put on the beryllocenes [15]. It was stated that the beryllium systems are less ionic than the magnesium analogues. The main-group metallocenophanes were also analyzed in a recent study [16]. It was found that the non-parallel structures occur not only for calcocenes, as was discussed in another study [14], but also in other species, as for example, in stannocene and plumbocene [16].

Very recently, the BP86-D3/TZ2P calculations were performed on half-sandwich and sandwich beryllium and magnesium moieties [17]. The results of the calculations show that beryllium and magnesium species are ruled mainly by electrostatic interactions; however, a covalent character of interactions is also pronounced. The latter is more evident for beryllium species than for magnesium ones. The decomposition energy calculations also show that the interactions of beryllium and magnesium cations with the cyclopentadienyl rings are stronger and “more covalent” than the corresponding Be-X and Mg-X interactions (where X designates a halogen center).

Interestingly, numerous studies have shown that the interactions of the first-group cations with other species, particularly with the π-electron systems, are more ionic than such interactions of the second-group cations (such as the beryllium and magnesium ones mentioned previously) [18]. Thus, the work presented here aims at furthering and deepening our understanding about the nature of the interactions in sandwich and sandwich-like complexes containing the cations of the first group.

## 2. Computational Details

**Figure 1:**
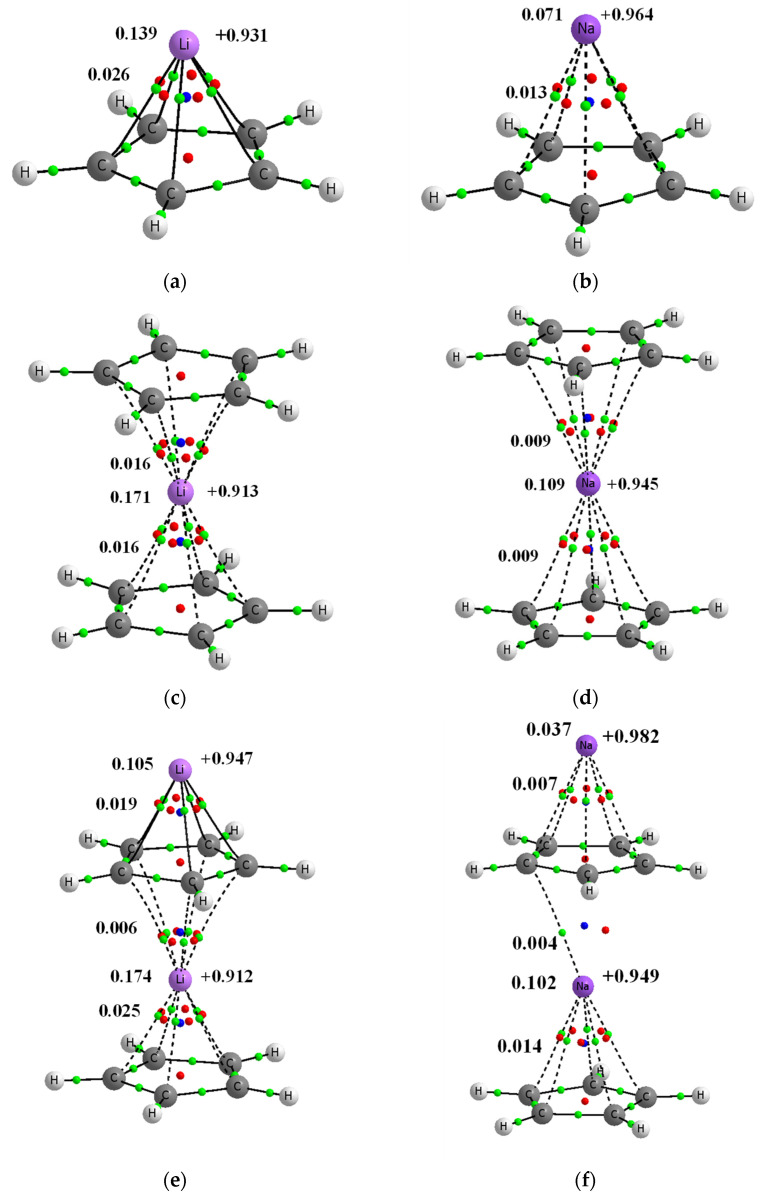

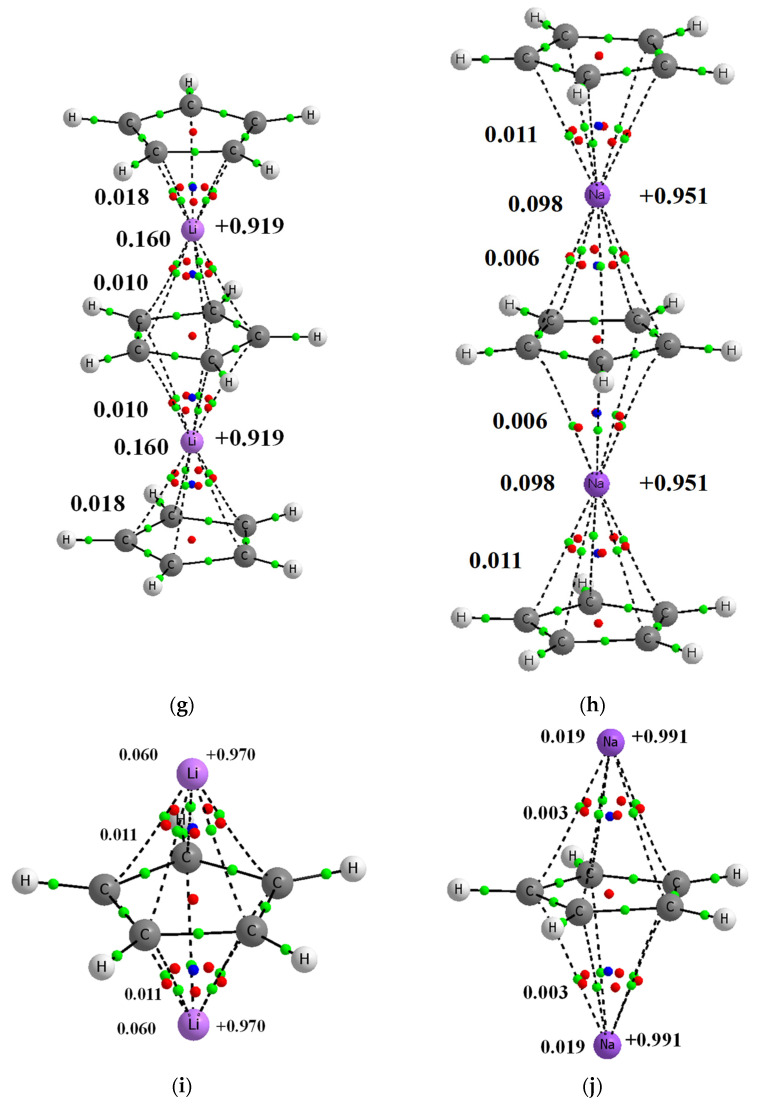
Molecular graphs of complexes’ analyzed here; big circles correspond to attractors and green small circles to bond critical points, while red and blue small circles correspond to ring and cage critical points, respectively. Continuous and broken lines indicate bond paths. Few characteristics of complexes are presented, NBO charges of lithium centers are presented at right sides of pictures, the Wiberg indices are given at the left side (of C∙∙∙Li^+^/Na^+^ contacts and of the whole Li^+^/Na^+^ centers). (**a**) Li^+^C_5_H_5_^−^ (**b**) Na^+^C_5_H_5_^−^ (**c**) C_5_H_5_^−^Li^+^C_5_H_5_^−^ (**d**) C_5_H_5_^−^Na^+^C_5_H_5_^−^ (**e**) Li^+^C_5_H_5_^−^Li^+^C_5_H_5_^−^ (**f**) Na^+^C_5_H_5_^−^Na^+^C_5_H_5_^−^ (**g**) C_5_H_5_^−^Li^+^C_5_H_5_^−^Li^+^C_5_H_5_^−^ (**h**) C_5_H_5_^−^Na^+^C_5_H_5_^−^Na^+^C_5_H_5_^−^ (**i**) Li^+^C_5_H_5_^−^Li^+^ (**j**) Na^+^C_5_H_5_^−^Na^+^.

The complexes that are composed of the cyclopentadienyl anions and lithium or sodium cations are the subject of this study. These are half-sandwich, sandwich, triple-decker and other sandwich-like systems, as can be seen in Figure 1. The calculations for these complexes, i.e., the geometry optimizations, were performed with the use of the Gaussian16 set of codes [19]. The ωB97XD functional [20] with the aug-cc-pVTZ basis set [21] were applied. It was justified that the ωB97XD functional offers more reliable results in comparison with other commonly applied functionals [20]. It was also shown that for the analysis of interactions, this functional, especially in conjunction with the aug-cc-pVTZ basis set, provides results superior to other functionals and basis sets [22]. Frequency calculations were carried out at the same ωB97XD/aug-cc-pVTZ level for all systems considered; imaginary frequencies were not found, confirming that the optimized systems correspond to energetic minima. For all complexes analyzed here that contain two or more cyclopentadienyl anions, the neighboring anions are twisted relative to each other, and thus these complexes may be classified as staggered ones. One may expect that for the systems discussed here there are various configurations with different symmetries. This is not a subject of the analysis here. Different configurations and different symmetries were analyzed for sandwich-like structures, but only for the simple systems containing two carbon-rings [14,15,16]. Larger linear and non-linear sandwich-like chains that occur in crystal structures are discussed only occasionally.

The BP86-D3/TZ2P level was applied to perform energy decomposition calculations for the ωB97XD/aug-cc-pVTZ geometry optimized complexes. That is, the BP86 functional [23,24] with the Grimme dispersion corrections [25] and the uncontracted Slater-type orbitals (STOs) as basis functions with triple-ζ quality for all elements [26] were applied. The energy decomposition calculations [27,28] were performed with the use of the ADF2013.01 program [28,29]. The total interaction energy in the energy partitioning applied here is composed of terms according to Equation (1) given below.

ΔE_int_ = ΔE_elstat_ + ΔE_Pauli_ + ΔE_orb_ + ΔE_disp_ (1)

The term ΔE_elstat_ is usually attractive (negative) and it corresponds to the quasi-classical electrostatic interaction between the unperturbed charge distributions of atoms. The Pauli repulsion, ΔE_Pauli_, is the energy change associated with the transformation from the superposition of the unperturbed electron densities of the isolated fragments to the wave function that properly obeys the Pauli principle through antisymmetrization and renormalization of the product wave function. The orbital interaction, ΔE_orb_, corresponds to the charge transfer and polarization effects, i.e., to electron charge shifts resulting from complexation.

The NBO method [30,31] was used to calculate the atomic charges as well as the energies of the most significant orbital–orbital interactions. In the A-H∙∙∙B hydrogen bonded systems, the n_B_ → σ_AH_^*^ overlap is the most important orbital–orbital interaction [30,31,32]. n_B_ is the lone electron pair of the B proton accepting center while σ_AH_^*^ marks an antibond orbital of the Lewis acid unit. The above-mentioned interaction energy is expressed by Equation (2).

∆E (n_B_ → σ_AH_^*^) = q_i_ 〈n_B_∣*F*∣ σ_AH_^*^〉^2^/(ε (σ_AH_^*^) − ε (n_B_)) (2)

〈n_B_∣*F*∣σ_AH_^*^〉 is the Fock matrix element, (ε (σ_AH_^*^) − ε (n_B_)) is the orbital energy difference and q_i_ is the donor orbital occupancy. In the case of the complexes analyzed in this study, two types of overlaps between orbitals of cyclopentadienyl anion and alkali metal cation are the most important: n_C_ → n_Li/Na_ and σ_cc_ → n_Li/Na_. However, for each complex analyzed, none of the interaction energy corresponding to such overlap exceeds 1 kcal/mol. The NBO 6.0 program [33] implemented in the ADF2019 set of codes [28,29] was used to carry out NBO calculations; this program was also applied to calculate atomic charges and the Wiberg indices [34].

The “Quantum Theory of Atoms in Molecules“, QTAIM [35,36], was applied to analyze characteristics of the bond critical points corresponding to interactions occurring in the structures analyzed in this study. The AIMAll program [37] was used to carry out QTAIM calculations. Systematic searches through the Cambridge Structural Database, CSD [38,39], were performed to find crystal structures containing the type of motifs that are the subject of this study.

## 3. Results and Discussion

### 3.1. Crystal Structures

The search through the Cambridge Structural Database, CSD [38,39], was performed to find decker sandwich-like crystal structures. The search was constrained to having at least two alkali metal cations located between cyclopentadienyl anions or other five-membered ring carbon structures. Accordingly, C_5_H_5_^−^∙∙∙A^+^∙∙∙C_5_H_5_^−^∙∙∙A^+^∙∙∙C_5_H_5_^−^ fragments (A = Li, Na, K, Cs, Fr), or other ones where the C_5_H_5_^−^ anion is replaced by species containing a five-member ring carbon motif, were searched for. The search was also constrained to distances between the A^+^ cation and two neighboring carbon species, C∙∙∙A, that were shorter than the sum of corresponding van der Waals radii. The radii that were proposed by Bondi [40] and that are inserted in the CSD were applied here. However, only two C∙∙∙A contacts are taken into account for each neighboring pair (as the C_5_H_5_^−^∙∙∙A^+^ pair, for example) and they concern non-adjacent carbon atoms of the five-membered carbon ring. The following criteria of accuracy for these searches were applied: 3D coordinates determined, no disordered structures, no errors, no polymeric structures, R-factor less or equal to 10% and only single crystal structures.

Twelve crystal structures were found in this search. In five of the structures, the metal ions located between carbon rings are the sodium cations, whereas in the remaining seven they are the potassium cations. However, whenever any of the criteria of accuracy were not applied then 29 crystal structures were found. Specifically, 4, 7 and 18 structures that contain lithium, sodium and potassium ions, respectively. In two crystal structures, the cesium cations occur apart from the lithium cations, and in another structure, the cesium cations occur apart from the potassium cations.

**Figure 2:**
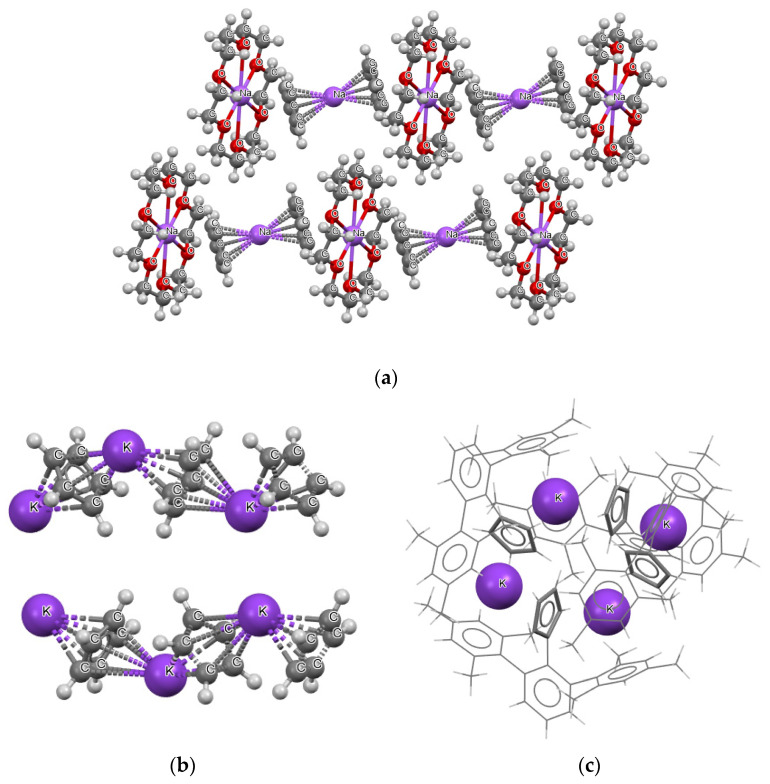
Three examples of crystal structures containing decker motifs: (**a**) (1,4,7,10,13,16-hexaoxacyclooctadecane)-sodium bis(η^5^-cyclopentadienyl)-sodium, (**b**) catena-((μ_2_-η^5^, η^5^-cyclopentadienyl)-potassium), (**c**) potassium 2-(1^2^,1^4^,1^6^,3^2^,3^4^,3^6^-hexamethyl [1^1^,2^1^:2^3^,3^1^-terphenyl]-2^2^-yl)cyclopentadienyl (for the simplicity of this picture, the structure is presented by sticks, except potassium cations).

Figure 2 shows examples of three crystal structures; in one of them (Figure 2a), (1,4,7,10,13,16-hexaoxacyclooctadecane)-sodium bis(η^5^-cyclopentadienyl)-sodium structure [41], the sodium cations are located between cyclopentadienyl anions. However, two types of arrangements of the sodium cations are apparent. In one type, there is no close atom–atom contacts apart from those between the sodium cations and the cyclopentadienyl rings. In another type of arrangement, there are additional close contacts of the sodium cations with the oxygen centers of the 1,4,7,10,13,16- hexaoxacyclooctadecane molecules.

Figure 2b presents another example, i.e., the crystal structure of catena-((μ_2_-η^5^, η^5^-cyclopentadienyl)-potassium), and the C_5_H_5_^−^∙∙∙K^+^∙∙∙C_5_H_5_^−^∙∙∙K^+^∙∙∙C_5_H_5_^−^ chains occur in this structure [42]. One may say that “infinite-type” decker structures occur here (in reality these chains are restricted by the size of crystals). The fragment of the crystal structure that shows two chains is presented in Figure 2b. Figure 2c presents the fragment of the crystal of potassium 2-(1^2^,1^4^,1^6^,3^2^,3^4^,3^6^-hexamethyl [1^1^,2^1^:2^3^,3^1^-terphenyl]-2^2^-yl) cyclopentadienyl [43]. One can see that another type of sandwich-like structure occurs here. Four cyclopentadienyl species and four potassium cations located alternately one to another form a ring.

The aforementioned three examples show that there are various types of crystal packing that realize the decker-type arrangements. In addition, regular parallel collocation of rings, which are interspersed with alkali cations, do not exist; the crystal packing disturbs such regular arrangements (see Figure 2b for an example where the anion–cation chains are not regular).

### 3.2. The Energetic Parameters

The interaction energies and the corresponding energy decomposition results, i.e., the terms of energy, for complexes analyzed in this study are presented in Table 1. The energy decomposition was performed according to the scheme briefly described in the former section (Equation (1)). The majority of complexes are treated as composed of two monomers. The division of the complex into monomers is designated in Table 1 by three points. This means that for the same complex various interactions may be taken into account. For example, for the C_5_H_5_^−^Li^+^C_5_H_5_^−^Li^+^C_5_H_5_^−^ complex, the interaction energy of the C_5_H_5_^−^Li^+^C_5_H_5_^−^Li^+^ unit with the C_5_H_5_^−^ anion is equal to −78.05 kcal/mol whereas for the same complex the interaction energy of the C_5_H_5_^−^Li^+^ species with the C_5_H_5_^−^Li^+^C_5_H_5_^−^ anion is −43.49 kcal/mol. It is worth mentioning that there is one exception from the above-mentioned division of complexes into two conjoint monomers. For the Li^+^C_5_H_5_^−^Li^+^ cation, the additional division into the C_5_H_5_^−^ anion and two Li^+^ cations is performed (the Li^+^∙∙∙C_5_H_5_^−^∙∙∙Li^+^ designation in Table 1). The strongest interaction was observed here (ΔE_int_ = −328.26 kcal/mol). A similar additional division is applied for the analogous sodium Na^+^C_5_H_5_^−^Na^+^ moiety where ΔE_int_ = −270.09 kcal/mol. Some important observations concerning the interactions of the complexes analyzed in this study follow next.

In general, the absolute values of the interaction energies, |ΔE_int_|’s, are greater for the lithium species than for their sodium analogues. For example, |ΔE_int_| for the Li^+^C_5_H_5_^−^∙∙∙Li^+^ interaction is equal to 64.97 kcal/mol, which is 8.49 kcal/mol larger than that for the Na^+^C_5_H_5_^−^∙∙∙Na^+^ analogue (56.48 kcal/mol). The only exception occurs for the C_5_H_5_^−^Li^+^∙∙∙C_5_H_5_^−^Li^+^ and C_5_H_5_^−^Na^+^∙∙∙C_5_H_5_^−^Na^+^ pair of interactions since |ΔE_int_| is respectively equal to 23.00 kcal/mol and 25.67 kcal/mol, i.e., a slightly stronger interaction was observed for the sodium complex than for the lithium analogue.

**Figure 3:**
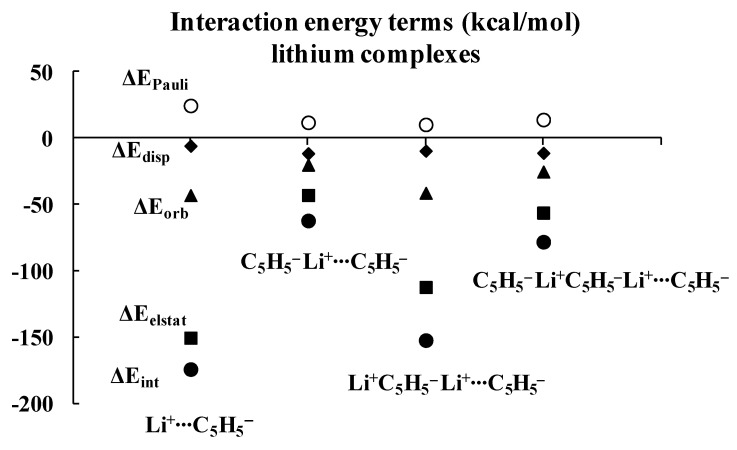
Energies of interactions (and their components according to Equation (1), all in kcal/mol) of a few complexes analyzed here; this figure presents the influence of the extension of the whole lithium system on these energies.

**Figure 4:**
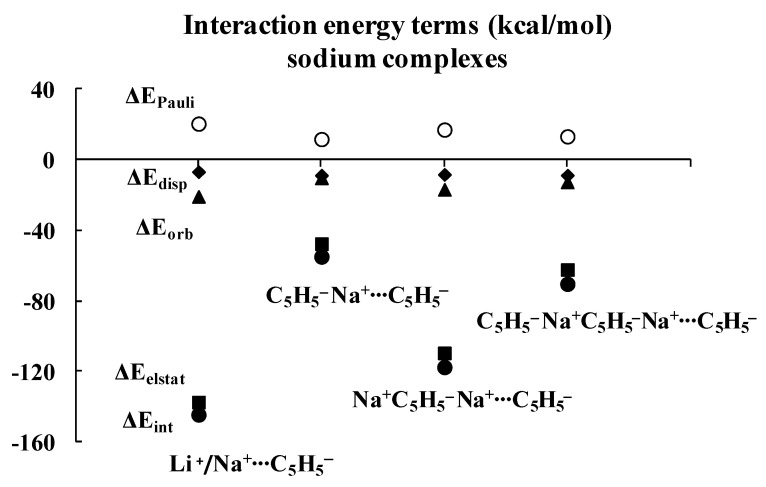
Energies of interactions (and their components according to Equation (1), all in kcal/mol) of a few complexes analyzed here; this figure presents the influence of the extension of the whole sodium system on these energies.

Regarding the lithium complexes, the strongest interaction was observed for the Li^+^C_5_H_5_^−^ complex, |ΔE_int_| = 174.17 kcal/mol. It is worth noting that when the size of the complex increases, the interaction between the cyclopentadienyl ring and the remaining part of the complex gets weaker. For example, the |ΔE_int_| value is 62.02 kcal/mol for the C_5_H_5_^−^Li^+^C_5_H_5_^−^ complex. Likewise, |ΔE_int_| value is equal to 152.30 kcal/mol for the Li^+^C_5_H_5_^−^Li^+^C_5_H_5_^−^ complex, and yet it is only 78.05 kcal/mol for the C_5_H_5_^−^Li^+^C_5_H_5_^−^Li^+^C_5_H_5_^−^ moiety. Similar changes were observed for the sodium analogues, with |ΔE_int_| values of 144.47, 54.88, 117.54 and 70.35 kcal/mol for the Na^+^C_5_H_5_^−^, C_5_H_5_^−^Na^+^C_5_H_5_^−^, Na^+^C_5_H_5_^−^Na^+^C_5_H_5_^−^ and C_5_H_5_^−^Na^+^C_5_H_5_^−^Na^+^C_5_H_5_^−^ complexes, respectively, whenever the interaction of the C_5_H_5_^−^ unit with the remaining part of the complex is considered. These systematic energetic changes for the lithium and sodium complexes discussed above are displayed in Figure 3 and Figure 4, respectively. One can see that, for these complexes, |ΔE_int_| exceeds 100 kcal/mol when the cation∙∙∙anion interactions are considered; in contrast, |ΔE_int_| is lower than 100 kcal/mol when the neutral unit∙∙∙anion interactions are considered. These results indicate an important role of the electrostatic term of interaction in the stabilization of such complexes.

Inspection of Table 1 shows that the ΔE_elstat_/ΔE_orb_ ratios are greater for cation∙∙∙anion interactions than for the corresponding ratios for neutral unit∙∙∙anion interactions in the two sets of four complexes (lithium and sodium) discussed previously. The terms of the energies of interactions are also shown in Figure 3 and Figure 4. Particularly, the changes for the electrostatic and orbital energy terms, ΔE_elstat_ and ΔE_orb_, follow similar trends as those seen for the total interaction energies, ΔE_int_’s, i.e., decrease, increase and decrease in absolute values of energies if the size of the complex increases.

Table 1 also shows that, in general, the electrostatic interaction is the most important attractive term for all complexes analyzed here, followed next by the orbital interaction, and last by the dispersion term. There are three exceptions, however, for which the dispersion term is actually larger than the orbital one: C_5_H_5_^−^Li^+^∙∙∙C_5_H_5_^−^Li^+^, C_5_H_5_^−^Na^+^∙∙∙C_5_H_5_^−^Na^+^ and C_5_H_5_^−^Na^+^∙∙∙C_5_H_5_^−^Na^+^C_5_H_5_^−^. It is worthwhile to note that two of these three complexes involve interactions between neutral units, and that all three complexes exhibit the weakest interactions among all complexes considered here, with |ΔE_int_| < 43 kcal/mol.

As mentioned above, the crucial role of the electrostatic interaction, ΔE_elstat_, was observed in all complexes analyzed since the corresponding term resulting from the decomposition shows that it is the most important contribution to the attraction interaction energy. The comparison of the ΔE_elstat_/ΔE_orb_ ratios shows that they are greater for the sodium complexes than for the corresponding lithium analogues, indicating that, in the former complexes, the electrostatic attraction is much more important than in the latter ones. In addition, this ratio is greater for the cation∙∙∙anion interaction than for the neutral unit∙∙∙anion interaction. For the lithium complexes, the lowest ratio for the cation∙∙∙anion interactions was observed for the Li^+^C_5_H_5_^−^Li^+^∙∙∙C_5_H_5_^−^ system, 2.73. All interactions with neutral units are characterized by the lower ratios. In the case of sodium complexes, the lowest ratio for the cation∙∙∙anion interactions was observed for the Na^+^C_5_H_5_^−^Na^+^∙∙∙C_5_H_5_^−^ system, 6.52. The interactions of one cyclopentadienyl anion with two cations are characterized by the greatest ratios, 5.39 and 12.81, for lithium and sodium species, respectively.

### 3.3. The Larger Chain Structures of Cyclopentadienyl Complexes

It was discussed in the previous section that the strongest interactions (characterized by the greatest |ΔE_int_| values) between the terminal cyclopentadienyl ring and the remaining part of the complex occur in both lithium and sodium series for the simplest Cat^+^⋅⋅⋅Cp^−^ complexes (Cat^+^ = Li^+^ or Na^+^, Cp^−^ = C_5_H_5_^−^). The enlargement of the latter systems results in the weakening of interactions. The subsequent addition of an anion weakens the interaction, and then the subsequent addition of the cation leads to its strengthening. It is expressed by Figure 3 and Figure 4, for the lithium and sodium complexes, respectively. However, as the complex increases by an anion or cation unit, the difference between interaction energies in complexes differing by only one-unit decreases. One may expect that the further enlargement of the complex results in the further diminishing of the latter difference.

To check this tendency, additional calculations were performed here for the systems occurring in Figure 3 and Figure 4 and for the corresponding greater systems for the less saturated Pople style 6-311++G(d,p) basis set [44]. So, the ωB97XD/6-311++G(d,p) level was applied. The complexes optimized at this level correspond to energetic minima since imaginary frequencies are not observed here. These are “linear systems”, which means the cations and the centers of cyclopentadienyl anions are located approximately at the same line (as occurs for ωB97XD/aug-cc-pVTZ complexes discussed so far). However, the closest anions, separated only by one cation, are twisted, forming a staggered structure. That occurs for all ωB97XD/6-311++G(d,p) complexes optimized here.

**Figure 5:**
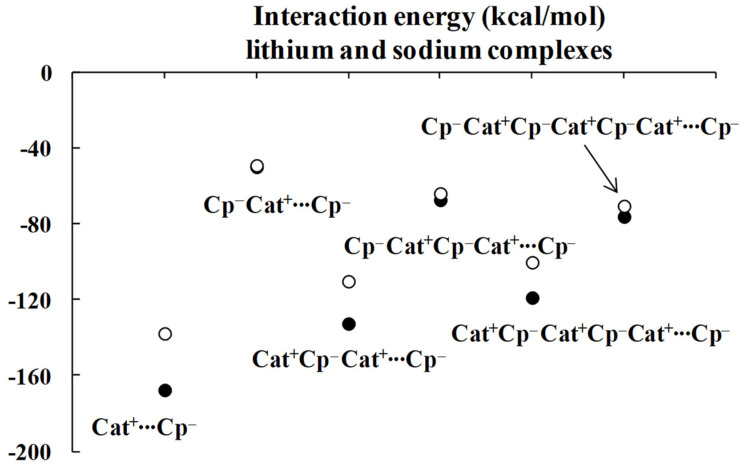
The ωB97XD/6-311++G(d,p) energies of interactions (in kcal/mol) of complexes analyzed here; the lithium species are designated by black circles while the sodium ones are designated by the open circles.

Table 2 presents the interaction energies corrected for the basis set superposition error, BSSE [45], and for complexes optimized at the ωB97XD/6-311++G(d,p) level, BSSE corrections are also specified there. The division of the complex into monomers, the terminal C_5_H_5_^−^ cation and the remaining part is designated as before by three points. One can see that the tendency of the decrease in the interacting energies´ difference between complexes differing only by one anion or cation is preserved for the larger systems. This tendency is also apparent in Figure 5, where the relationships between the size of complex and the interaction energy for lithium and sodium series are presented.

One can expect that for larger systems containing more Cp^−^ and Cat^+^ units, the further adding of cations and anions does not change the interaction energies for the specified cyclopentadienyl anion. It is worth mentioning that the chains discussed here correspond to arrangements that occur in numerous crystal structures, where large chains of sandwich-like structures are observed.

### 3.4. The QTAIM and NBO Results

The Quantum Theory of Atoms in Molecules, QTAIM [35,36], characteristics as well as other results derived from the Natural Bond Orbital, NBO, method [30,31] are discussed in this section. Table 3 presents the cation∙∙∙carbon distances (upper lines) and the electron densities, ρ_BCP_´s, at bond critical points, BCPs (bottom lines), of the corresponding bond paths (see also Figure 1).

For each C_5_H_5_^−^∙∙∙Li^+^/Na^+^ pair in a contact, the mean C∙∙∙Li^+^/Na^+^ distance and the mean ρ_BCP_ value are shown. However, for such pairs, the differences between the C∙∙∙Li^+^/Na^+^ distances do not exceed 0.001 Å, and the differences between corresponding ρ_BCP_ values are also negligible. The Na^+^C_5_H_5_^−^Na^+^C_5_H_5_^−^ complex is slightly different since the differences between C∙∙∙Na^+^ distances for the “middle” C_5_H_5_^−^∙∙∙Na^+^ pair are greater, up to 0.005 Å. This is a special kind among the anion–cation neighboring pairs since only one C∙∙∙Na^+^ bond path occurs here (see Figure 1). The mean C∙∙∙Na^+^ distance is equal to 2.822 Å here. Moreover, for this contacting pair, the weakest interaction was observed when compared with the other sodium complexes, where |ΔE_int_| is equal to 25.67 kcal/mol.

**Figure 6:**
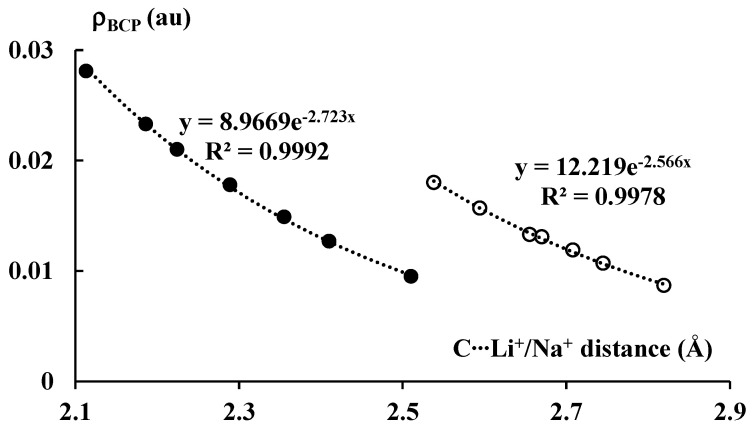
The exponential relationships between the C∙∙∙Li^+^/Na^+^ distance (in Å) and the electron density (in au) at BCP of the corresponding bond path. The lithium and sodium species are marked by the black and white circles, respectively.

Figure 6 presents exponential correlations between the carbon–cation distance and the electron density at BCP of the corresponding bond path. Two separate correlations, for lithium and sodium complexes, are presented. It is worth mentioning that the ρ_BCP_ value is often treated as a measure of the strength of interaction. Numerous correlations between the electron density at BCP and other measures of the strength of interaction were reported in other studies [46], particularly in the case of hydrogen bonded systems [47,48]. It was also reported in numerous studies that the atom–atom distance often roughly expresses the strength of the corresponding interaction [49]. Thus, the correlations presented in Figure 6 may be treated as those between measures of the strength of interaction.

It is interesting that the ρ_BCP_ values for the systems analyzed here correlate with the energies presented in Table 1, i.e., the stronger the interactions, the greater the corresponding ρ_BCP_ values that are observed. One can also see that the ρ_BCP_ values for the lithium systems are greater than for the corresponding sodium analogues; the former complexes are linked by stronger interactions than the latter ones. It should be noted that, for all complexes discussed here, the |ΔE_int_| values are relatively large, with no values below 20 kcal/mol and several values over 100 kcal/mol. Notwithstanding these relatively large |ΔE_int_| values, low values of ρ_BCP_ for the C∙∙∙Li^+^/Na^+^ contacts occur.

It has been shown that the ρ_BCP_ parameters, which often correlate with the total interaction energies, are rather related to the covalent character of interactions [50]. This is in agreement with the results presented here for complexes analyzed. The Quantum Theory of Atoms in Molecules, QTAIM, characteristics indicate that interactions discussed here may be only slightly covalent in nature. The low ρ_BCP_ values for these interactions were already pointed out above. The Laplacian of the electron density at BCP, ∇^2^ρ_BCP_, is positive as well as the total electron energy density at BCP, H_BCP_, for all carbon–cation contacts. The latter two parameters, ∇^2^ρ_BCP_ and H_BCP_, would confirm the covalent character of an interaction considered if they were negative [51,52].

**Figure 7:**
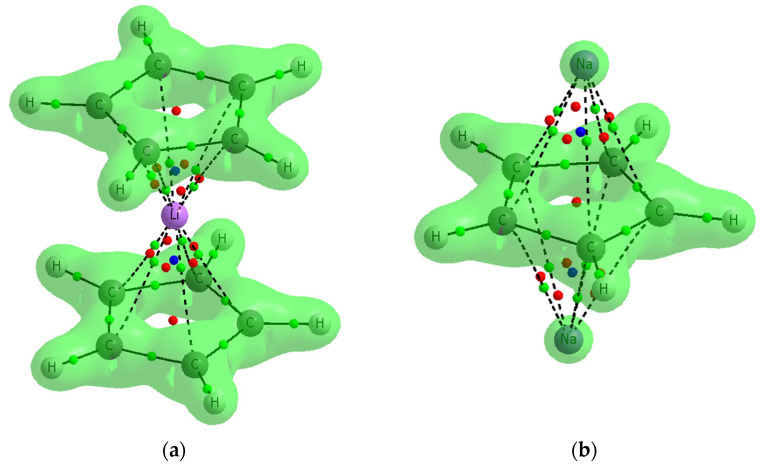
The examples of two complexes analyzed here where contours of the ∇^2^ρ = 0 surfaces are presented: (**a**) C_5_H_5_^−^Li^+^C_5_H_5_^−^ (**b**) Na ^+^C_5_H_5_^−^Na^+^.

Figure 7 presents two examples, the C_5_H_5_^−^Li^+^C_5_H_5_^−^ and Na^+^C_5_H_5_^−^Na^+^ complexes with the surfaces characterized by the Laplacian equal to zero, ∇^2^ρ = 0. The areas closed by those surfaces are characterized by the negative ∇^2^ρ; thus, the interactions of pairs of linked atoms within them are covalent in character. This figure shows that the interactions between cyclopentadienyl rings and cations are rather not covalent in nature, the BCPs of the corresponding C∙∙∙Li^+^/Na^+^ contacts are in areas of the positive ∇^2^ρ. This observation is true also for all other complexes analyzed in this study.

**Figure 8:**
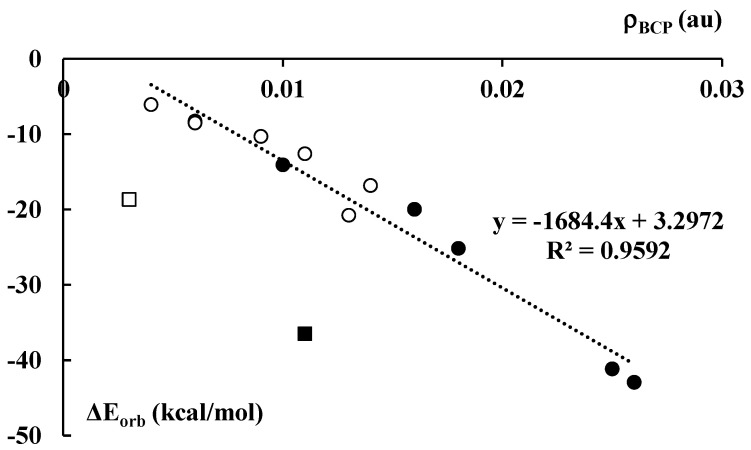
The dependence between the electron density (in au) at the C∙∙∙Li^+^/Na^+^ BCP of the corresponding bond path and the orbital–orbital interaction energy term (Equation (1), in kcal/mol) for the corresponding cation–cyclopentadienyl anion contact. Black and white circles correspond to the lithium and sodium species, respectively. Black and white squares not included in the correlation presented correspond to the Li^+^C_5_H_5_^−^∙∙∙Li^+^ and Na^+^C_5_H_5_^−^∙∙∙Na^+^ species, respectively.

The orbital energy, ΔE_orb_, in the decomposition scheme (Equation (1)) is often related to the covalent character of interaction since it corresponds to the electron charge shifts resulting from complexations. Figure 8 presents a fairly good linear correlation between the electron density at BCP, ρ_BCP_, of the C∙∙∙Li^+^/Na^+^ contact and the orbital interaction energy, ΔE_orb_. The cation–anion pair that corresponds to the latter contact indicates a way of the complex partition into interacting units. This correlation is valid for all lithium and sodium complexes, except of the Li^+^C_5_H_5_^−^Li^+^ and Na^+^C_5_H_5_^−^Na^+^ ones. They are excluded from this correlation since they differ from other species discussed here. For example, only in these cases do the number of cations overwhelms the number of anions. Accordingly, the corresponding interactions for the latter complexes differ from those occurring in the remaining systems.

**Figure 9:**
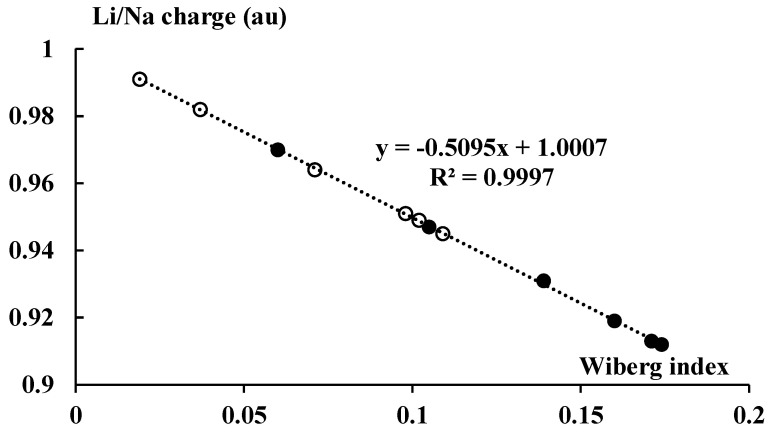
The linear correlation between the total Wiberg index of the Li or Na center and the corresponding NBO charge (in au). The lithium and sodium species are marked by the black and white circles, respectively.

Figure 9 presents the NBO charges of cations that occur in the complexes discussed here (they are also presented in Figure 1).

For the lithium systems, the Li-charge is within the 0.91–0.97 au range, whereas for the sodium systems the charge is within the 0.95–0.99 au range. Accordingly, negligible electron charge shifts take place upon complexation in the systems considered here; the cation charges are close to unity, even closer in the case of the sodium species. This means that the orbital interaction energy, ΔE_orb_, should be less important than the electrostatic one for the systems analyzed here. It is less important for the sodium complexes than for the lithium ones since the ΔE_elstat_/ΔE_orb_ ratio is greater for the former species than for the latter ones.

Figure 1 also presents the Wiberg indices for the cation–carbon contacts, mean values for each pair of the cyclopentadienyl anion and the neighboring cation. The Wiberg index is approximately related to the bond order and consequently to the strength of interaction [34,53]; the total Wiberg indices for cations are also presented in Figure 1. The total Wiberg indices are the sums of all Wiberg indices related to links between the cation and other centers (in the case of this study these are the hydrogen and carbon centers). Figure 9 presents an excellent linear correlation between the NBO charge of the cation and its total Wiberg index, the greater the charge (closer to unity) then the lower the value of the Wiberg index. It means that the greater outflow of the electron charge from the ligands to the linked cation results in the greater bond orders of this cation. It should lead to the formation of bond orbitals in extremal cases of such electron charge shifts. This is not observed for the systems analyzed here, given the slight electron charge shifts that occur.

## 4. Summary

The Cambridge Structural Database, CSD, searches show that in the majority of systems containing alternately cyclopentadienyl anion and the first group of periodic system cation, the sodium cations occur most often, followed by the potassium cations, and the occurrence of lithium cations is rather rare.

The results of calculations presented here show that in the systems containing lithium or sodium cations between cyclopentadienyl anions, the electrostatic interaction energy is the most important attractive contribution to the total attractive interaction energy. The orbital interaction is less important; it is related to the electron charge shifts that are not important in systems analyzed here. The charges of cations, lithium or sodium ones, are close to unity in complexes analyzed, consistent with negligible electron charge shifts resulting from complexations.

The interactions between the cyclopentadienyl rings and the cations are mostly not covalent in nature; the covalent character is only slightly manifested. The QTAIM characteristics confirm the electrostatic nature of such interactions since, for the bond critical points corresponding to the cation–carbon bond paths, the Laplacian of the electron density and the total electron energy density are positive. It is interesting that the electron density at the cation–carbon bond critical point, ρ_BCP_, expresses the strength of the local interaction. The latter value is in agreement with the interaction energy. However, a better correlation was observed between the orbital energy, ΔE_orb_, and ρ_BCP_. The Wiberg index reflects the negligible electron charge shifts between cations and the cyclopentadienyl anions.

It is worth noting that the relationships and parameters presented and discussed in this study may be applied for a broader class of systems, as for example, for those containing the cations of the first group of the periodic system. For example, the lithium salt of the alumole dianion was discussed and the X-ray crystal structure analysis of this system shows that two lithium cations are located at both sites of the planar AlC_4_ ring [54]. A similar situation was observed for the X-ray crystal structure of dilithioplumbole, where two lithium cations are situated above and below the planar plumbole ring [55]. These two lithium structures are very similar to the Li^+^C_5_H_5_^−^Li^+^ system analyzed in this study, where large interactions are detected.

In this study, all results show the dominance of the electrostatic interaction in complexes analyzed. However, the covalent character, much less important than electrostatic one, was also observed, although it is negligible. It is more pronounced in lithium structures than in the sodium ones. Figure 6 shows the greater electron density at the bond critical point, ρ_BCP_, and the shorter Li⋅⋅⋅C distance for the lithium systems than for the corresponding sodium ones, the ρ_BCP_ value and the Na⋅⋅⋅C distance, respectively. Figure 8 shows greater orbital energies, |ΔE_orb_|, for Li-complexes than for Na-complexes. Finally, Figure 9 presents the charges of Li and Na species that are lower for lithium complexes. The latter corresponds to greater Wiberg indices that are related to the sum of bond orders of the center considered. The observed Wiberg indices are greater for the lithium systems than for the sodium ones.

## Figures and Tables

**Table 1:** The interaction energy terms (in kcal/mol) according to the scheme expressed by Equation (1); the ΔE_elstat_/ΔE_orb_ ratio is also included. The decomposition scheme was applied for the lithium and sodium species for divisions of complexes indicated by three points.

System	ΔE_int_	ΔE_Pauli_	ΔE_elstat_	ΔE_orb_	ΔE_disp_	ΔE_elstat_/ΔE_orb_
Li-complexes						
Li^+^∙∙∙C_5_H_5_^−^	−174.17	24.79	−150.47	−42.92	−5.58	3.51
C_5_H_5_^−^Li^+^∙∙∙C_5_H_5_^−^	−62.02	12.06	−42.83	−19.96	−11.29	2.15
Li^+^C_5_H_5_^−^Li^+^∙∙∙C_5_H_5_^−^	−152.30	10.48	−112.24	−41.16	−9.37	2.73
C_5_H_5_^−^Li^+^C_5_H_5_^−^Li^+^∙∙∙C_5_H_5_^−^	−78.05	14.17	−56.09	−25.15	−10.98	2.23
C_5_H_5_^−^Li^+^∙∙∙C_5_H_5_^−^Li^+^	−23.00	8.57	−12.70	−8.26	−10.61	1.54
C_5_H_5_^−^Li^+^∙∙∙C_5_H_5_^−^Li^+^C_5_H_5_^−^	−43.49	11.56	−29.53	−14.08	−11.45	2.10
Li^+^C_5_H_5_^−^∙∙∙Li^+^	−64.97	14.06	−34.80	−36.47	−7.76	0.95
Li^+^∙∙∙C_5_H_5_^−^∙∙∙Li^+^	−328.26	33.36	−292.19	−54.20	−15.22	5.39
Na-complexes						
Na^+^∙∙∙C_5_H_5_^−^	−144.47	20.48	−137.39	−20.78	−6.78	6.61
C_5_H_5_^−^Na ^+^∙∙∙C_5_H_5_^−^	−54.88	11.72	−47.55	−10.28	−8.78	4.63
Na^+^C_5_H_5_^−^Na^+^∙∙∙C_5_H_5_^−^	−117.54	17.11	−109.54	−16.80	−8.32	6.52
C_5_H_5_^−^Na^+^C_5_H_5_^−^Na^+^∙∙∙C_5_H_5_^−^	−70.35	13.26	−62.23	−12.60	−8.78	4.94
C_5_H_5_^−^Na ^+^∙∙∙C_5_H_5_^−^Na ^+^	−25.67	6.60	−17.74	−6.07	−8.46	2.92
C_5_H_5_^−^Na^+^∙∙∙C_5_H_5_^−^Na^+^C_5_H_5_^−^	−42.45	9.25	−34.30	−8.51	−8.89	4.03
Na ^+^C_5_H_5_^−^∙∙∙Na^+^	−56.48	10.90	−40.77	−18.67	−7.94	2.18
Na^+^∙∙∙C_5_H_5_^−^∙∙∙Na^+^	−270.09	26.93	−261.02	−20.37	−15.63	12.81

**Table 2:** The interaction energies calculated at the ωB97XD/6-311++G(d,p) level (in kcal/mol) for the lithium and sodium complexes; BSSE corrections are included (kcal/mol).

System	Li Complexes		Na Complexes	
	ΔE_int_	BSSE	ΔE_int_	BSSE
Cat^+^∙∙∙Cp^−^	−167.5	0.6	−137.7	0.8
Cp^−^Cat^+^∙∙∙Cp^−^	−49.9	1.1	−49.1	1.0
Cat^+^Cp^−^Cat^+^∙∙∙Cp^−^	−132.5	1.2	−110.2	1.0
Cp^−^Cat^+^Cp^−^Cat^+^∙∙∙Cp^−^	−67.3	1.2	−63.9	1.0
Cat^+^Cp^−^Cat^+^Cp^−^Cat^+^∙∙∙Cp^−^	−118.8	1.2	−100.2	1.0
Cp^−^Cat^+^Cp^−^Cat^+^Cp^−^Cat^+^∙∙∙Cp^−^	−76.1	1.1	−70.5	1.0

**Table 3:** The C∙∙∙Li^+^/Na^+^ distances (upper, mean values for each contact between the cation and the cyclopentadienyl anion, in Å) and the mean electron density (bottom values, in au) of the BCPs of corresponding carbon–cation bond paths, ρ_BCP_. The contacts are presented in the same order as for the designations given in the first column, i.e., from the left site to the right site.

Complex	C∙∙∙Li^+^/Na^+^ Distance (Up) and ρ_BCP_ (Down)			
Li^+^C_5_H_5_^−^	2.113 0.0281			
C_5_H_5_^−^Li^+^C_5_H_5_^−^	2.355 0.0149	2.354 0.0150		
Li^+^C_5_H_5_^−^Li^+^C_5_H_5_^−^	2.128 0.0269	2.510 0.0095	2.186 0.0233	
C_5_H_5_^−^Li^+^C_5_H_5_^−^Li^+^C_5_H_5_^−^	2.289 0.0178	2.410 0.0127	2.409 0.0127	2.289 0.0178
Li^+^C_5_H_5_^−^Li^+^	2.224 0.0210	2.224 0.0210		
Na^+^C_5_H_5_^−^	2.538 0.0180			
C_5_H_5_^−^Na ^+^C_5_H_5_^−^	2.708 0.0119	2.708 0.0119		
Na^+^C_5_H_5_^−^Na^+^C_5_H_5_^−^	2.568 0.0166	2.822 0.0087	2.594 0.0157	
C_5_H_5_^−^Na^+^C_5_H_5_^−^Na^+^C_5_H_5_^−^	2.670 0.0131	2.745 0.0107	2.745 0.0107	2.670 0.0131
Na ^+^C_5_H_5_^−^Na^+^	2.655 0.0133	2.650 0.0133		

## Data Availability

Not applicable.

## Sample Availability

Not available.
